# Longitudinal genetic studies of cognitive characteristics

**DOI:** 10.18699/VJ20.599

**Published:** 2020-02

**Authors:** R.N. Mustafin, A.V. Kazantseva, R.F. Enikeeva, S.B. Malykh, E.K. Khusnutdinova

**Affiliations:** Bashkir State Medical University, Ufa, Russia; Institute of Biochemistry and Genetics – Subdivision of the Ufa Federal Research Centre of the Russian Academy of Sciences, Ufa, Russia; Institute of Biochemistry and Genetics – Subdivision of the Ufa Federal Research Centre of the Russian Academy of Sciences, Ufa, Russia; Psychological Institute of the Russian Academy of Education, Moscow, Russia M.V. Lomonosov Moscow State University, Laboratory of psychology of professions and conflicts, Moscow, Russia; Institute of Biochemistry and Genetics – Subdivision of the Ufa Federal Research Centre of the Russian Academy of Sciences, Ufa, Russia M.V. Lomonosov Moscow State University, Laboratory of psychology of professions and conflicts, Moscow, Russia

**Keywords:** brain, cognitive functions, longitudinal studies, transposable elements, головной мозг, когнитивные характеристики, лонгитюдные исследования, транспозоны

## Abstract

The present review describes longitudinal studies of cognitive traits and functions determining the causes of their variations and their possible correction to prevent cognitive impairment. The present study reviews the involvement of such environmental factors as nutrition, prenatal maternal stress, social isolation and others in cognitive functioning. The role of epigenetic factors in the implementation of environmental effects in cognitive characteristics is revealed. Considering the epigenome significance, several studies were focused on the design of substances affecting methylation and histone modification, which can be used for the treatment of cognitive disorders. The appropriate correction of epigenetic factors related to environmental differences in cognitive abilities requires to determine the mechanisms of chromatin modifications and variations in DNA methylation. Transposons representing stress-sensitive DNA elements appeared to mediate the environmental influence on epigenetic modifications. They can explain the mechanism of transgenerational transfer of information on cognitive abilities. Recently, large-scale meta-analyses based on the results of studies, which identified genetic associations with various cognitive traits, were carried out. As a result, the role of genes actively expressed in the brain, such as BDNF, COMT, CADM2, CYP2D6, APBA1, CHRNA7, PDE1C, PDE4B, and PDE4D in cognitive abilities was revealed. The association between cognitive functioning and genes, which have been previously involved in developing psychiatric disorders (MEF2C, CYP2D6, FAM109B, SEPT3, NAGA, TCF20, NDUFA6 genes), was revealed, thus indicating the role of the similar mechanisms of genetic and neural networks in both normal cognition and cognitive impairment. An important role in both processes belongs to common epigenetic factors. The genes involved in DNA methylation (DNMT1, DNMT3B, and FTO), histone modifications (CREBBP, CUL4B, EHMT1, EP300, EZH2, HLCS, HUWE1, KAT6B, KMT2A, KMT2D, KMT2C, NSD1, WHSC1, and UBE2A) and chromatin remodeling (ACTB, ARID1A, ARID1B, ATRX, CHD2, CHD7, CHD8, SMARCA2, SMARCA4, SMARCB1, SMARCE1, SRCAP, and SS18L1) are associated with increased risk of psychiatric diseases with cognitive deficiency together with normal cognitive functioning. The data on the correlation between transgenerational epigenetic inheritance of cognitive abilities and the insert of transposable elements in intergenic regions is discussed. Transposons regulate genes functioning in the brain due to the processing of their transcripts into non-coding RNAs. The content, quantity and arrangement of transposable elements in human genome, which do not affect changes in nucleotide sequences of protein encoding genes, but affect their expression, can be transmitted to the next generation.

## Abstract (ru)

Рассмотрена роль лонгитюдных исследований когнитивных характеристик в определении причин, влияющих на познание, с целью возможной их коррекции для улучшения познавательных навыков. В данных исследованиях показано, что на развитие когнитивных функций влияют такие средовые факторы, как качество нутриентов, стресс во время гестации и характер социального окружения. Выявлены специфические эпигенетические изменения, выступающие в качестве посредников между генотипом и средой в реализации когнитивных функций. В связи с важным значением эпигенома перспективна разработка методов терапии когнитивных расстройств с использованием агентов, влияющих на метилирование и модификации гистонов. Развивающимся направлением в этой области является изучение некодирующих РНК, которые способны модифицировать эпи- генетические факторы. За последние годы проведены широкомасштабные метаанализы результатов исследова- ний роли генетических ассоциаций с различными когнитивными характеристиками. Показано значение активно экспрессирующихся в головном мозге генов, таких как BDNF, COMT, CADM2, CYP2D6, APBA1, CHRNA7, PDE1C, PDE4B, PDE4D. С физиологическим познанием оказались ассоциированы гены, вовлеченные в развитие психических заболеваний (MEF2C, CYP2D6, FAM109B, SEPT3, NAGA, TCF20, NDUFA6). В развитие психических заболеваний с ког- нитивным дефицитом вовлечены гены, участвующие в метилировании ДНК (DNMT1, DNMT3B, FTO), модификации гистонов (CREBBP, CUL4B, EHMT1, EP300, EZH2, HLCS, HUWE1, KAT6B, KMT2A, KMT2D, KMT2C, NSD1, WHSC1, UBE2A) и моделировании хроматина (ACTB, ARID1A, ARID1B, ATRX, CHD2, CHD7, CHD8, SMARCA2, SMARCA4, SMARCB1, SMARCE1, SRCAP, SS18L1), которые имеют значение в регуляции когнитивных функций у здоровых людей. Приведены дан- ные, позволяющие предположить, что трансгенерационное наследование когнитивных характеристик связано с некодирующими РНК, а также со способностью мобильных элементов, инсертированных в межгенные области, влиять на регуляцию функционирующих в головном мозге генов за счет процессинга транскриптов транспозо- нов в некодирующие РНК. Особенности состава, количества и распределения в геноме мобильных элементов, которые не изменяют нуклеотидные последовательности белок-кодирующих генов, но влияют на их экспрес- сию, могут передаваться из поколения в поколение.

## Stability and variability of cognitive abilities in ontogenesis

Recently, in addition to highly informative methods of molecular biological research used for the identification of specific genetic loci involved in cognitive functioning at the genomewide level, studies on the detection of genetic determinants under the longitudinal paradigm have become of great importance. Longitudinal studies make it possible to obtain specific objective data on dynamics and to evaluate the contribution of genetic and environmental factors to the changes in cognitive differences in human ontogenesis. Cognitive abilities include information perception (gnosis), speech, intelligence, memory, attention and praxis (motor skills) (Medaglia et al., 2015), spatial perception ability, vocabulary, information processing speed and executive functioning. Specific cognitive tests together with multivariate genetic analysis were used to assess them (Plomin, Deary, 2015)

The results of meta-analyses of longitudinal studies demonstrated that genetic factors (Bergen et al., 2007; Haworth et al., 2010; Franić et al., 2015), environmental influences (Wong et al., 2010), and age (Briley, Tucker-Drob, 2013; Tucker-Drob, Briley, 2014) significantly affected cognitive development. The average changes in cognitive abilities during individual development were reported to increase significantly from infancy to adolescence, and gradually decrease in adulthood (Tucker-Drob, Briley, 2014). One of the first reports on a rapid increase in the longitudinal stability of cognitive abilities from infancy to adolescence was reported by Bayley (1949). This study demonstrated a significant variability in general intelligence in early childhood, which achieved relative stability by school age (Bayley, 1949). The data presented were confirmed by a meta-analysis based on longitudinal studies using objective cognitive tests (Tucker-Drob, Briley, 2014). This study examined the following cognitive abilities: general intelligence, active vocabulary, verbal and nonverbal abilities (including IQ), selective and constant attention, working and spatial memory, visual attentiveness, and substitution of digital symbols. The results obtained in 15 independent longitudinal samples revealed low to moderate correlations between genetic component and common (shared) environment and cognitive abilities in early childhood, while they increased sharply and achieved a high level by adolescence until the late adulthood. The correlations between individual environment were low in childhood and gradually increased to moderate in adulthood. Interestingly, an enhanced phenotypic stability of cognitive abilities in child development was almost entirely mediated by genetic factors (Tucker-Drob, Briley, 2014).

A wide range of population and ontogenetic variability of various cognitive abilities was demonstrated. For different cognitive characteristics the average coefficient of genetic correlation was 0.6, while phenotypic correlation was 0.3. The highest level of heritability was reported for general intelligence (factor “g”) – varying from 40 % in childhood to 80 % in adults. The scholastic Assessment Test and American College Test were used to measure these parameters (Zabaneh et al., 2018). The changes in each of the cognitive abilities during individual development are specific, with a unique contribution of environmental and genetic components. For example, the impact of common environment was 0.21 and of heritability coefficient was 0.51 for mathematical abilities measured by individual’s ability to read and study mathematics using a combination of network tests. At the same time, these values for reading ability (measured using the Reading Efficiency Test (TOWRE), one of four tests from the TEDS analysis) were 0.14 and 0.66, respectively (Davis et al., 2014).

Among all cognitive abilities, the study of intelligence is highly significant, since validated tests estimating standard IQ indicators are used. For example, an individual with IQ < 50 is diagnosed with severe intellectual disability (ID), affecting 0.4 % of the population. About half of ID cases are observed in chromosomal and monogenic diseases (Kleefstra et al., 2014). Assortative mating accumulates genetic variance in the population in each generation, thus contributing to an additive genetic variance of intelligence. Intelligence out of mental psychopathology is normally distributed with a positive result of an exceptional characteristic representing a model for “positive genetics” (Plomin, Deary, 2015). Heritability of intelligence varies significantly depending on the studied population. For example, estimates of IQ heritability in twin studies in Russia appeared to be higher than in comparable studies from the USA. This observation is due to the similarity in living conditions of individuals from Russia. IQ heritability varies depending on socio-economic status; it is significantly higher in high-income families. The difference in IQ among African Americans and European Americans in the United States was about one standard deviation (15 points of IQ) in the 20th century, although recently it appeared to be decreased (Sternberg, 2012).

Molecular genetic studies play an important role in assessing ontogenetic variability in cognitive characteristics. In 2007, a meta-analysis of six longitudinal studies examining the role of hereditary factors in cognitive differences based on two or more time intervals to minimize age variability was conducted. An increasing contribution of heritability in cognitive abilities was revealed from 13 (55 %) to 25 (70 %) years, which evidences a significance of interactions between the genotype and the environment (Bergen et al., 2007). In a 2010, a meta-analysis involving 11,000 twin pairs demonstrated an enhanced heritable component in general cognitive abilities from 41 % at 9 years to 55 % at 12 years and 66 % at 17 years. General cognitive abilities were assessed using the Stanford-Binet Intelligence Scale, including vocabulary measurement, pattern analysis, memorizing sentences and numbers, quantitative tests (Haworth et al., 2010). The heritability of intelligence linearly increases from 20 % in the infancy to 40 % in adolescence and 60 % in adulthood, with its maximum of 80 % in the elderly and further decreasing to 60 % after 80 years. Genome-wide quantitative trait analysis and twin studies reported different levels of heritability for certain cognitive abilities: 35 and 47 % for intelligence, respectively, 16 and 59 % for reading, 32 and 48 % for mathematical abilities, 35 and 41 % for language skills (Plomin, Deary, 2015).

An increasing impact of the genetic component in cognitive abilities from infancy to adolescence can be explained by amplified and innovative effects in infancy. A large-scale meta-analysis based on the results of 16 longitudinal studies examining the role of genetic and environmental components in cognitive functioning in 11,500 pairs of twins and siblings assessed twice within the period from 6 months to 18 years, revealed that in early childhood innovative adaptation effects prevail as a response to novel environmental stimuli and rapidly decrease by adolescence. The amplified effects characterizing the transfer of the influence of factors that were active in infancy to the subsequent stages in ontogenesis are amplified with further development. To measure cognitive characteristics in these studies, tests for intelligence and objective knowledge were used (Briley, Tucker-Drob, 2013).

What are the mechanisms underlying individual differences in cognitive abilities in ontogenesis? Some researchers suggest that the stability in cognitive functioning over time is due to the consistent exposure to the same exogenous environmental factors. Therefore, the stability of cognitive abilities reflects social, educational and economic stability. From another point of view, the stability of individual differences in cognitive abilities in ontogenesis is due to the continuous effect of endogenous factors (genes), while exogenous influences are irregular and have unstable effects. Thus, exogenous and endogenous factors, contributing to overall stability at different degrees differentially affect cognitive functions with age (Tucker-Drob, Briley, 2014).

## Genetic studies of cognitive functions

The results of the genome-wide association study (GWAS) of cognitive abilities established several associations, and polygenic estimates account for about 1 % of the variance in cognitive functions. Different studies evidence a small effect of each genetic variant in cognitive development. However, polygenic score, which accumulates the effects of single DNA variants to predict a genetic predisposition for each individual, can be estimated (Zabaneh et al., 2018). Several studies reported associations of alleles with cognitive abilities, which may represent the basis for further experimental analysis on the possible targeted effects on the products of these genes. From a clinical point of view, the study of neurotransmitter systems’ genes in specific cognitive functions are of most interest, since it would help propose a pharmacotherapy of cognitive impairment from the existing drugs.

Genetic studies of individual cognitive abilities have been carried out to identify the role of certain genes in cognitive development. The association analysis of SNPs with cognitive abilities such as memory, educational background, and verbalnumerical abilities, revealed the involvement of genes that play an important role in brain development and functioning. These genes include CADM2 (encodes a synaptic cell adhesion protein in the central nervous system), CYP2D6 (encodes a cytochrome metabolizing serotonin and neurosteroids) and APBA1 (encodes a protein that interacts with the amyloid precursor in Alzheimer’s disease). Verbal-numeric abilities were measured using a 13-point survey presented on a touch screen computer. Memory was measured using the “pair matching” task: participants observed a random grid of 12 cards with six pairs of matching characters for 5 seconds. To measure educational preparation, individuals were asked the question “Which of the following qualifications do you have?” followed by a list of possible answers (Davies et al., 2016). In 2014, Das et al. observed significant main and interaction effects of COMT and BDNF genotypes on reaction time (Das et al., 2014). Alleles of the COMT gene are also associated with cognitive functions such as executive cognition and cognitive control (measured using prefrontal tasks). The association of alleles of the CHRNA7 gene (encodes alpha-7 receptor of the nicotinic subunit) with attention gating was detected – the measurement was performed using H50 ERP (even-related potential, which occurs in the temporal limbic cortex) (Goldberg, Weinberger, 2004). In 2019, a meta-analysis carried out with the inclusion of 1.1 million mentally healthy individuals confirmed the allelic association of the BDNF gene and phosphodiesterases PDE1C, PDE4B, PDE4D with differences in cognitive traits such as educational level and mathematical abilities. The measurement was performed using normalized cognitive test scores (Gurney, 2019).

In healthy individuals, associations of genes involved in the development of psychiatric disorders with cognitive impairments were identified. GWAS was conducted involving 78,308 people, and 336 SNPs were confirmed to be associated with cognitive functions. This study detected the involvement of genes associated with Alzheimer’s disease (MEF2C and EXOC4 genes) and schizophrenia (MEF2C, CYP2D6, FAM109B, SEPT3, NAGA, TCF20, and NDUFA6 genes). The measurement of fluid intelligence was carried out by various questionnaires (“touch screen” or “web interface”) with the number of correct answers of 13 questions (Sniekers et al., 2017). Cognitive impairment is comorbid to both mental and behavioral disorders. For example, intelligence impairment is observed in attention deficit hyperactivity disorder (ADHD) (Claesdotter et al., 2018). According to scientific data, ADHD is associated with genes responsible for the normal cognitive functioning. These genes include DRD4, SLC6A3 (Junkiert- Czarnecka, Haus, 2016), and 5-HTTLPR (Owens et al., 2012). Their research is promising to clarify the mechanisms affecting gene networks involved in neurotransmitter systems functioning in normal and brain pathology cases. The commonality of genetic architecture of cognitive abilities and disabilities was assumed. Hence, the data on cognitive pathology can possibly be used for the study of cognitive abilities. It was also revealed that genes involved in variations in normal intelligence are associated with ID. According to the analysis of the OMIM database, about half of all human genetic diseases have a neurological component, which frequently comprises ID (Crabtree, 2013).

Molecular genetic studies of cognitive abilities and disabilities (Franić et al., 2015) confirm the “generalist genes hypothesis” proposed by Professor Robert Plomin (Plomin, Kovas, 2005). According to this hypothesis, the same set of genes significantly affects different areas of cognitive functioning. In addition, individual variations and changes in general cognitive traits including reading and linguistic abilities tend to be mutually correlated, which indicates a commonality in their etiology (Chow et al., 2013).

Cognitive impairments (CI) represent a heterogeneous group of diseases, which have been actively studied. The general mechanisms of these diseases together with the molecular processes underlying human cognition are identified. A significant role in these processes belongs to the genes encoding the proteins involved in epigenetic regulation. They participate in brain development and maintenance, necessary for adaptation to changing physical and social conditions. These genes were reported to be involved in both normal cognitive development and CIs with a pronounced genetic liability to autism spectrum disorders, ID, intellectual retardation, and schizophrenia. Fifty five genes with epigenetic influence were identified. They are divided into four categories: (1) writers, (2) erasers, (3) chromatin remodelers of the DEAD/H-ATPase family, and (4) other readers and chromatin remodelers. The writers include the genes involved in DNA methylation (DNMT1, DNMT3B, FTO) and involved in the addition of amino acid residues to side groups of histones (CREBBP, CUL4B, EHMT1, EP300, EZH2, HLCS, HUWE1, KAT6B, KMT2A, KMT2D, KMT2C, NSD1, WHSC1, and UBE2A). The lateral groups are molecules that attach to the central carbon atom of an amino acid residue, thus changing its biochemical properties. Therefore, the binding between histones and DNA molecules is either enhanced or weakened. The erasers include the HDAC4, HDAC8, KDM5C, KDM6A, and PHF8 genes. The products of these genes remove the lateral histone groups. Chromatin remodeling genes of the DEAD/H-ATPase family involved in the regulation of the nucleosome position include the ACTB, ARID1A, ARID1B, ATRX, CHD2, CHD7, CHD8, SMARCA2, SMARCA4, SMARCB1, SMARCE1, SRCAP, and SS18L1 genes. Other chromatin readers and remodulators include ASXL1, BCOR, CHMP1, CTCF, GATAD2B, HCFC1, KANSL1, MBD5, MECP2, PHF6, POGZ, SKI, MED12, MED17, MED23, NIPBL, RAD21, SALL1, SMC1A, and SMC3. The role of these genes in the etiology and pathogenesis of several CIs was revealed (Kleefstra et al., 2014), which can represent the basis for future research into the possible correction of ID using target therapy due to reversible nature of epigenetic modifications.

## Epigenetic regulation of cognitive functions

Epigenetic mechanisms that are central in brain development, structure and functioning can affect changes in cognitive traits in ontogenesis, since differences in gene expression are age- and cell-type specific (Dauncey, 2014). For example, a violation of epigenetic regulation is observed in cognitive aging as a result of changes in DNA methylation, expression of non-coding RNAs (ncRNAs), and post-translational modification of histones (Mather et al., 2014). Epigenetic mechanisms include DNA methylation, histone modifications, ATP-based chromatin remodeling complexes, Polycomb-Tritorax protein complexes, ncRNAs, potential prions, transcription factor binding and other mechanisms involved in the formation and maintenance of the inherited chromatin structure and its attachment to the nuclear matrix (Bell, Spector, 2011). Epigenetic processes represent a reversible regulation of various genomic functions. They are necessary for tissue differentiation and long-term regulation of gene functions. Their dynamic changes are caused by many factors including environmental influences, variations in DNA sequences, and stochastic events (Wong et al., 2010).

The study of the influence of hereditary and environmental factors on changes in DNA methylation is promising. Quantitative measurements of DNA methylation in the promoter regions of the dopamine D4 receptor (DRD4), serotonin transporter (SLC6A4) and monoamine oxidase A (MAOA) genes were performed using DNA samples of 46 pairs of monozygotic and 45 dizygotic twins aged 5 to 10 years (Wong et al., 2010). The association of gene alleles and cognitive abilities was identified (Owens et al., 2012; Junkiert-Czarnecka, Haus, 2016). It was found that differences in DNA methylation appeared even in early childhood in genetically identical individuals and were unstable with time. The results of longitudinal studies obtained suggest that environmental influences are important factors of individual changes in DNA methylation and differentially affect genomic structure. The observation of dynamic changes in DNA methylation over time underlines the importance of longitudinal studies of epigenetic factors (Wong et al., 2010). The analysis of DNA methylation of more than 27,000 CpG sites in the genome of 387 individuals aged from 1 to 102 years (in frontal and temporal cortex, pons and cerebellum) showed a positive correlation between age and DNA methylation in different brain structures. Moreover, CpG islands, which demonstrated a pronounced constant correlation between DNA methylation and chronological age, were identified (Hernandez et al., 2011). These results evidence that environmental factors have higher effects on DNA methylation in children compared to adults (Lupu et al., 2012).

During learning and memory formation, a dynamic regulation of the chromatin structure occurs in response to neuronal stimulation. Learning-induced chromatin changes include histone modifications such as acetylation, phosphorylation and methylation. Moreover, non-histone proteins are involved in chromatin modification, which play an important role in the regulation of transcriptional activity of neurons during memory consolidation. These proteins include the subunit p65/ RelA of the NF-kB DNA binding complex, the transcription factor p53, estrogen receptor alpha (ERα), DNA methyltransferase (DNMT1), tubulin, histone deacetylase (HDAC1), the glucocorticoid receptor, histone acetyltransferase p300/ CBP Associated Protein (Rudenko, Tsai, 2014).

The role of environmental influences including nutrition, xenobiotics, stress in pre- and postnatal periods in cognitive development requires the involvement of epigenetic mechanisms in gene expression regulation during brain functioning (Fine, Sung, 2014). Nutrition can cause brain changes in ontogenesis, comprising significant changes in cognitive functioning up to dementia. This effect is mediated by modified expression of many genes, while individual nutritional sensitivity depends on genetic variability. Thus, nutrition has an immediate and lasting effects on the epigenome. For example, micronutrients such as folate, vitamins B6 and B12, choline and methionine are involved in DNA methylation (Dauncey, 2014).

Other important environmental factors affecting the regulation of cognitive functions include exposure to opioids and other toxic substances in the prenatal period. A longitudinal study of children exposed to toxic substances demonstrated significant consequences even after 1, 2, 3, 41/2, 81/2 years, which represented a reduced IQ level compared to the control group of children (Nygaard et al., 2015). The prenatal exposure to toxic substances affected cognitive development of children due to changes in epigenetic profile. In particular, the results from ADHD children indicate a correlation of paracetamol intake in pregnancy for more than 20 days with changes in the methylation profile at more than 1600 CpG islands (Gervin et al., 2017). Maternal smoking during gestation is associated with specific methylation of selected regions of the AHRR (aryl-hydrocarbon receptor repressor) and CYP1A1 (cytochrome P450, family 11, subfamily A, polypeptide 1) genes in their children with ADHD in the postnatal period (Sengupta et al., 2017).

Longitudinal studies reported that children subjected to prenatal stress in the early stages of development were characterized by a lower development rate and decreased cognitive performance in the first year of life (if stress and increased cortisol levels were present at the early stages of prenatal development). However, an elevated maternal cortisol level at the end of pregnancy was associated with higher cognitive development and performance at the age of 12 months. These results suggest that maternal cortisol and pregnancy-specific anxiety have a programmed effect on the developing fetus, which can be mediated by epigenetic factors (Davis, Sandman, 2010). Social isolation in early childhood causes differential cognitive development via an epigenetic effect on the expression of genes involved in brain functioning, such as the BDNF gene (Li et al., 2016).

Thus, published findings indicate a crucial role of epigenetic factors in cognitive development in ontogenesis. Each individual demonstrates a unique epigenetic response to environmental stimuli, which manifests in an individual level of cognitive abilities. Therefore, the question as to the mechanisms of transgenerational transfer (especially, paternal) of epigenetic regulation of cognitive functions arises. It can be assumed that transposable elements (TEs), which play an important role in the regulation of epigenetic processes, can be attributed to the structures involved in the transfer of the cognitive level to next generations (Mustafin, Khusnutdinova, 2017). It was confirmed by transgenerational epigenetic programming of individual personality traits from parents who experienced a severe environmental stress to their F3 and F4 generation (Savvateeva-Popova et al., 2015). This observation can be explained by TE stress-sensitivity, since novel germinative insertions including stress reaction are transmitted to descendants (Mustafin, Khusnutdinova, 2019). TE location in the genome is reflected in their site-specific integrations under various factors, which specifically affect neurogenesis (Feng et al., 2013; Fujiwara, 2015). It can be explained by TE influence on the expression of genes differentiating in hippocampal neuronal stem cells (NSC) (Jacques et al., 2013; Gerdes et al., 2016). Indeed, high activity of TEs (Faulkner, 2011; Kurnosov, 2015) and their transfer under stress is cellspecific (Hunter et al., 2012). These effects are associated with genomic plasticity (Muotri et al., 2005; Singer et al., 2010) and cognition (Aimone et al., 2014; Pastuzyn et al., 2018), which are mediated by TE interaction with epigenetic factors, including ncRNAs (Kapusta et al., 2013; Samantarrai et al., 2013; Zhang et al., 2015).

Changes in epigenetic regulation imply the absence of structural rearrangements in the genome, since it mainly comprises histone modifications, RNA interference, and DNA methylation. TEs can influence these mechanisms without modifying nucleotide sequences in exons, but exerting their regulatory effect on gene expression via intergenic inserts (de Souza et al., 2013; Chuong et al., 2017; Barry, 2018). Ontogenetically, these properties contribute to tissue-specific differentiation of cells (Trizzino et al., 2018). With respect to hippocampal neurogenesis, the highest TE activity can be associated with epigenetic reprogramming of gene transcriptional activity for functional remodeling of differentiated neurons (Faulkner, 2011; Kurnosov et al., 2015; Upton et al., 2015). Changes in the expression of the majority of LTR-containing TEs (endogenous retroviruses) were detected in mice by prenatal administration of valproic acid. It may explain a transgenerational effect of this drug on the delayed development of the nervous system and autism spectrum disorders (Tartaglione et al., 2019). An important role in the regulatory effect of TEs belongs to the processing of their transcripts to ncRNAs (Yuan et al., 2010, 2011; Qin et al., 2015).

A transgenerational transfer of epigenetic regulation of maternal cognitive abilities was based on stress (Braun et al., 2017; Misra, Ganesh, 2018) and alcohol exposure of the developing fetus (Doehner et al., 2017; Abbott et al., 2018). The changes are observed in F2 generation, since epigenetic transformation of the genome occurs in gametes in the prenatal period. The ncRNAs represent the most likely factors affecting transgenerational transfer of cognitive abilities (Daxinger, Whitelaw, 2012; Bohacek, Mansuy, 2015). At least 40 % of all long ncRNAs are expressed in the human brain, of which, for example, KCN2AS, BC1/200, BDNF, GDNF, EPHB2, KCNA2, are involved in the regulation of synaptic plasticity (Briggs et al., 2015; Pereira Fernandes et al., 2018). Changes in synaptic connections depending on individual experience are known as synaptic plasticity, which plays an important role in cognitive development (Woldemichael, Mansuy, 2016). Transcripts of long ncRNAs can be processed in miRNAs, which play an important role in the development of cognitive abilities (Barry, 2014). It was shown that dynamic changes in miRNA levels affect the expression of genes involved in cognitive development such as memory and learning (Woldemichael, Mansuy, 2016). The miRNAs interact with more than 90 % of synaptical proteins (Woldemichael, Mansuy, 2016).

The role of miRNAs in the transgenerational transmission of cognitive abilities may be associated with their influence on neuronal differentiation by changing the expression profile of certain genes (Stappert et al., 2015). The miRNA levels are specific in certain types of neurons (Smirnova et al., 2005). MiR-134 is involved in memory regulation by affecting CREB expression (Gao et al., 2010). Prolonged expression of miR-132 causes cognitive deficiency by inhibiting acetylcholinesterase activity (Shaltiel et al., 2013); miR-182 suppresses long-term memory by interacting with actin-regulatory proteins (Griggs et al., 2013); miR-124 affects learning and memory by regulating mRNA expression of GTPase-activating protein gene (IQGAP1) (Yang et al., 2014). MiR-2113 (Andrews et al., 2017), miR-151a-3p, miR- 212-3p, miR-1274b (Mengel-From et al., 2018) expression levels are associated with cognitive functioning. The study of epigenetic factors in the transgenerational transmission of cognitive abilities is promising for the development of preventive technologies of cognitive impairment in the next generations. Empirical use of the natural resveratrol analogue phytoalexin by female mice prevented cognitive dysfunctions in F1 and F2 generations due to changes in signaling pathways and epigenetic factors (Izquierdo et al., 2019).

## Conclusion

To assess the ontogenetic variability in cognitive abilities, the molecular genetic studies with a longitudinal design of the obtained data have been conducted. Longitudinal studies proved that an increasing phenotypic stability in cognitive abilities in human development was mediated by genetic factors. Higher impact of the heritable component in cognitive development varied from 41 % in children aged 9 years to 70 % in 25-year-old individuals. In early childhood, the prevalence of innovative adaptation effects on environmental factors was revealed, whereas heritability level depends on the examined cognitive ability. The association of CADM2, CYP2D6, and APBA1 gene alleles with memory consolidation, educational background, and verbal-numerical abilities was identified. Moreover, allelic variants of the BDNF and COMT genes are associated with reaction time; CHRNA7, with attention gating; and BDNF, PDE1C, PDE4B, PDE4D, with educational level and mathematical abilities. In addition, an association of the genes, previously demonstrated to be involved in the development of mental disorders (MEF2C, EXOC4, CYP2D6, FAM109B, SEPT3, NAGA, TCF20, NDUFA6), was determined with cognitive functioning in mentally healthy individuals. In the study of genes associated with cognitive impairment, the role of genes involved in epigenetic regulation (including DNA methylation, histone modification, and chromatin remodeling) was established.

During the last years, the study of the effect of epigenetic factors in cognitive differences appeared to be important, since they mediate the effect of environmental factors on cognition due to the chromatin regulation in dynamics. Epigenetic modifications can demonstrate an immediate and a long-term effect, both at the postnatal and prenatal periods. An important role in these effects is played by changes in DNA methylation at specific loci. It is assumed that transgenerational transmission of cognitive abilities was caused by TEs. This is due to their intergenic distribution and effect on the expression of specific ncRNAs. The importance of microRNAs for cognitive development suggests the possibility of their use as biomarkers and targets for potential therapeutic agents.

## Conflict of interest

The authors declare no conflict of interest.
